# The performance and assessment of hospital trauma teams

**DOI:** 10.1186/1757-7241-18-66

**Published:** 2010-12-13

**Authors:** Andrew Georgiou, David J Lockey

**Affiliations:** 1 Specialist Registrar in Anaesthesia & Intensive Care Medicine, Frenchay Hospital, Bristol BS16 1LE, UK; 2 Consultant in Anaesthesia & Intensive Care Medicine, Frenchay Hospital, Bristol BS16 1LE, UK

## Abstract

The purpose of the trauma team is to provide advanced simultaneous care from relevant specialists to the seriously injured trauma patient. When functioning well, the outcome of the trauma team performance should be greater than the sum of its parts. Trauma teams have been shown to reduce the time taken for resuscitation, as well as time to CT scan, to emergency department discharge and to the operating room. These benefits are demonstrated by improved survival rates, particularly for the most severely injured patients, both within and outside of dedicated trauma centres. In order to ensure the best possible performance of the team, the leadership skills of the trauma team leader are essential and their non-technical skills have been shown to be particularly important. Team performance can be enhanced through a process of audit and assessment of the workings of the team and the evidence currently available suggests that this is best facilitated through the process of video review of the trauma resuscitation. The use of human patient simulators to train and assess trauma teams is becoming more commonplace and this technique offers a safe environment for the future education of trauma team staff.

Trauma teams are a key component of most programmes which set out to improve trauma care. This article reviews the background of trauma teams, the evidence for benefit and potential techniques of performance assessment. The review was written after a PubMed, Ovid, Athens, Cochrane and guideline literature review of English language articles on trauma teams and their performance and hand searching of references from the relevant searched articles.

## Introduction

Trauma is the leading cause of death in the 1-44 year old age group [1] and the fourth leading cause of death in the western world [2]. Despite the widespread recognition of simple principles of trauma care which have the potential to reduce mortality and the implementation of trauma education initiatives such as the American College of Surgeons Advanced Trauma Life Support courses (ATLS^®^) [3], the uptake and implementation of many of these principles has been sporadic and variable. In the UK for example, The Royal College of Surgeons of England highlighted important deficiencies in the management of severely injured patients in a report in 1988 [4]. A second report in 2000 [5] addressed the lack of ongoing improvement in the last six years of the twentieth century [6], recommending amongst other things, the introduction of a system of trauma audit and the establishment of hospital trauma teams. In 2007 a report by the UK National Confidential Enquiry into Patient Outcomes and Death [2] found that trauma teams were only available in 20% of hospitals, and a trauma team response was documented for only 59.7% of patients with injury severity scores (ISS) >16. The report strongly recommended that hospitals in the UK ensure that a trauma team is available twenty four hours a day, seven days a week. This problem is not confined to the UK. Data from Australia in 2003 show that only 56% of adult trauma hospitals [7] and 75% of tertiary paediatric hospitals which receive trauma [8] provided a trauma team reception.

The trauma team usually comprises a multidisciplinary group of individuals drawn from the specialties of anaesthesia, emergency medicine, surgery, nursing and support staff, each of whom provide simultaneous inputs into the assessment and management of the trauma patient, their actions being coordinated by a team leader. The primary aims of the team are to rapidly resuscitate and stabilise the patient, prioritise and determine the nature and extent of the injuries and prepare the patient for transport to the site of definitive care, be that within or outside the receiving hospital. This 'horizontal' approach to trauma care aims to provide rapid input to a critically injured patient without the need to contact and request the presence of individual team members. This aims to reduce the time from injury to critical interventions and surgery. The original aim of the trauma team was to reduce the second peak of the trimodal distribution of death following trauma, by appropriately managing correctable disturbances to the airway, breathing and circulation, which, if well implemented, was predicted to reduce preventable deaths by 42% [9]. The validity of the trimodal concept has since been questioned [10,11] but the likely benefits of coordination and rapid assessment of the trauma victims by a trauma team are widely accepted.

### The Structure of the Trauma Team

**Figure 1:**
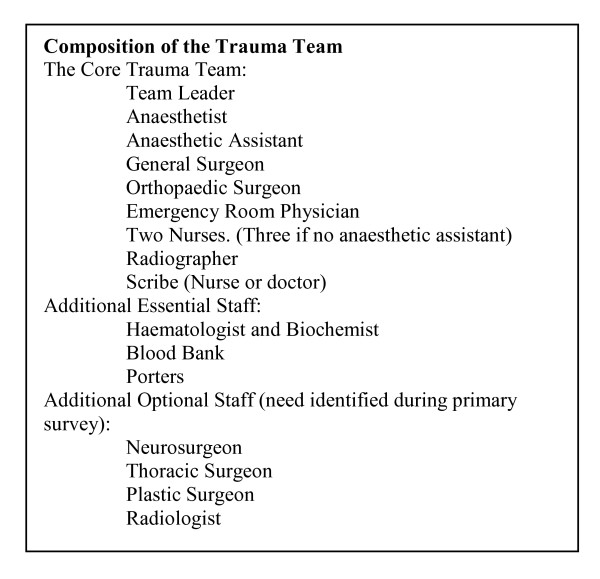
**The typical composition of a trauma team**. (Adapted from http://www.trauma.org[12]).

A typical trauma team composition is shown in Figure 1[12]. It is important not to over-staff the trauma team; excessive numbers of people in the core team can lead to fragmentation, with individuals failing to adhere to the directions of the team leader. Additional team members do not necessarily improve team function [13]. There are wide regional and national variations in the composition of hospital trauma teams and there has been much work in assessing the optimal makeup and performance dynamics of the trauma team. The presence of a surgeon on the trauma team is considered by some to be essential. The availability of an attending trauma surgeon on the trauma team twenty four hours a day has been demonstrated to reduce resuscitation time and time to incision for emergency operations, but has not been demonstrated to impact on mortality [14]. Many centres now have a tiered trauma team response according to the severity of injury of the trauma patient. The application of triggering systems attempts to ensure that the appropriate tier of trauma team response is activated. The triggering system usually depends on the reported mechanism of trauma, the assessed injuries or the derangement in physiology noted on examination [15-17]. Information from pre-hospital care providers is useful for guiding the appropriate tier of response and for assembly and preparation of the trauma team [18]. Although these triggering systems serve as useful guide as to when the team should be activated, a considerable rate of over-triage, in the region of 30 to 50%, is deemed essential to prevent any under-triage and therefore delays in mobilising the team where it is deemed essential [19].

The leader of the trauma team must be experienced in the diagnosis and management of trauma patients and the likely pitfalls associated with dealing with severely injured patients. This individual must also be comfortable directing and being responsive to other team members. Non technical skills such as leadership are particularly important [20]; a good team leader will change his leadership style according to the experience of the team and the severity of the trauma [21]. Commonly the leader is an emergency physician, a surgeon or an intensivist-anaesthetist. Data comparing surgeons with other trauma team leaders such as emergency physicians, show no difference in the length of stay in the emergency department or in the actual or predicted survival of patients [22,23]. The seniority of the physician present has been linked to team performance [24] and is a key feature of trauma system development [2].

### The Benefits and Pitfalls of a Trauma Team

Trauma systems have been shown to reduce mortality amongst the victims of trauma [25-29]. The trauma system is a multifaceted approach to trauma care involving professionals of many disciplines acting both pre-and in-hospital, within an organised model of care. The trauma team represents only one facet of the trauma system and separating the relative merits or drawbacks of the trauma team in isolation of the trauma system is not straightforward.

Data from Canada identifies that the involvement of the trauma team for patients with injury severity scores (ISS) >12 results in significantly better outcomes than if patients are dealt with on a service-by-service basis [30]. Not only was performance better than predicted, but there were more unexpected survivors in the group managed by the trauma team. Patients managed by a trauma team had higher ISS scores, were older, with more motor vehicle collisions and received more secondary transfers from other (non-trauma centre) hospitals, all of which should adversely affect the outcomes from this group, making the impact of the trauma team perhaps even more noteworthy. The incorporation of several specialties into one team therefore appears to be more valuable in outcome terms than the sum of its parts. The introduction of a trauma team in a level I trauma centre has been shown to reduce overall trauma mortality rates from 6.0% to 4.1% (absolute risk reduction 1.9%; 95% confidence interval 0.7%-3.0%), and in those severely injured patients with ISS scores >25, from 30.2 to 22.0% (absolute risk reduction 8.3%; 95% confidence interval 2.1%-14.4%) [31]. Data shows that the trauma team also improves survival in hospitals not recognised as trauma centres [32].

Trauma teams also reduce times from emergency department arrival to CT scan, to the operating room and to emergency department discharge, manifesting as improved survival amongst critically injured paediatric patients. The mortality benefit is however lost in paediatric patients who have less severe injuries [33]. Conversely, those patients who meet well established trauma call criteria, but who are not treated by the trauma team (i.e. the team was not called) have a higher mortality; 28% of all trauma patients fell into this category in a study of 2539 consecutive patients from China [34]. Part of the benefit of the trauma team may be related to a reduction in time to definitive care (often haemorrhage control). When well organised, the trauma team has been shown to reduce total resuscitation time from 122 to 56 minutes [35]. The introduction of a trauma team and a trauma service led to a ten fold reduction (4.3% to 0.46%) in delayed injury diagnosis in the setting of paediatric trauma in Salt Lake City [36], but the exact contribution of the trauma team to this improvement is not clear.

Despite the huge associated socioeconomic burden of increased morbidity no data on the impact of the trauma team on morbidity exist. It is clearly very difficult to separate the impact of a trauma team on morbidity and isolate it from the care received from scene to hospital discharge - a lengthy and variable pathway for many severely injured patients.

The initial phase of hospital care in the emergency room has been identified as the area where most preventable problems in trauma care occur [37]. The trauma team is naturally implicated in many of these errors. Common problems include errors or delays in treatment, diagnosis, and intervention. Inadequate system capacity and poor processes are also frequently implicated. Data from Australia identify that 6.09 errors per fatal case occur in the emergency department with an alarming 3.47 errors directly contributing to patient death [38].

In paediatric trauma resuscitation, 5.9 errors per case have been shown to occur but with no fatalities directly attributable to the resuscitation phase [39]. Emergency room problems, errors or inadequacies are however less likely to occur in a trauma centre where 1.7 errors occurred per case as opposed to 5.1 per case in small regional hospitals (p < 0.05) [37].

Interestingly, errors seem more common before 8 pm when staffing levels and expertise are usually greatest [40]. Such errors are likely due to failure to perform therapeutic or diagnostic measures at the right time, with the correct frequency or in the right order [38]. Unfamiliarity with the trauma scenario, disorganization of staff or equipment, failure to prioritise or realise the complexity of the problem, fixation error or misdiagnosis [38] all contribute to what is a critical time in the passage of the patient through the trauma system. Errors in communication are estimated to occur in more than 50% of trauma resuscitations [41], and this together with inadequate documentation, were the main reasons for trauma team leaders underperforming [42].

### Assessment of Trauma Team Performance

Evidence from the Scottish Trauma Audit Group has showed that the implementation of a trauma service audit programme can significantly improve survival in trauma patients. Survival rates for seriously injured trauma patients increased from 65 to79% through the course of the audit process, during which 53,000 trauma patients were seen in emergency departments in Scotland [43]. Assessment of the impact and performance of the trauma team as an isolated component of the trauma pathway is complex. Separation of the impact of multiple members of staff in a rapidly evolving environment with multiple variables is challenging and the optimal outcome measure that should be employed is open to debate.

Recording of error rates is somewhat crude and correlation of rates to outcome is fraught with confounding factors including assessor subjectivity and casemix variation. Assessment of single interventions rarely addresses the performance of a coordinated resuscitation attempt by professionals from different backgrounds. Carefully selected key performance indicators (e.g. time to CT scan) can be used to improve performance and set standards. Alternative outcomes may include compliance to local or published protocols [3], missed injury rates, improved outcomes and preventable deaths, all of which have benefits and drawbacks.

The optimal method of data acquisition during trauma team assessment has yet to be established. The options commonly employed are video review, observer review, medical notes review or the use of simulation. The remainder of this review will discuss the role for each.

### Video

Video review of trauma team resuscitation has been shown to identify more errors than review of the medical notes. The retrospective review of medical notes has been shown to miss 80% of resuscitation errors identified through video review [39]. Video has been shown to be a more efficient use of review time which allows correction of conceptual as well as technical errors. Errors identified by video analysis are most commonly those relating to the airway, breathing, provision of oxygen and omissions in the secondary survey [39]. In the analysis of tracheal intubation in trauma, video review was able to identify performance errors such as failure of team coordination; poor communication, and omission of key tasks by team members. Poor recovery from errors has also been identified [44]. These findings have led to revised practices to improve the safety of tracheal intubation in trauma [44].

Careful scrutiny of the video data may yield further details of the resuscitation attempt which may prove difficult to obtain by other means. For example, team leader performance [45], time to procedural intervention [40,46], compliance with ATLS guidelines [47] and assessment of the use of universal precautions [40] have all been examined by video review in the past. Video has also allowed assessment of process errors and reasoning which were found to occur in every case, although they were only infrequently judged to result in adverse outcomes. However errors of omission were judged to be more severe [41]; these include failure to consider, observe or document, available relevant information in order to select appropriate care. This was found to occur at a frequency of 2.4 errors per case [48]. Video review has identified that poor team organisation results in a significant increase in error, whereas adequate pre-hospital report, evident and efficient leadership, continued supervision of the patient, resuscitation in the correct order and working to defined protocols were each related to a lower total number of errors [40].

Review of videotaped trauma scenarios allows an appropriate source of feedback, debrief and learning for those concerned. In one study video review reduced the time to definitive care over a 3 month period by 13 minutes [49]. It has also allowed a retrospective review of the assessment of priorities during the resuscitation, the cognitive and physical integration of the workup by the team leader, team member adherence to assigned responsibilities, resuscitation time, errors or breaks in technique and behaviour change over time [49]. Through this process of performance review and retrospective learning, resuscitations have been shown to become more efficient and adherence to assigned responsibilities have improved [49]. Video data collection can be used to provide a quality appraisal system, for example during out-of-hours care, where no supervisor is available on site. The process of video review of trauma resuscitations therefore has benefits of performance and error analysis, audit and education, which together may manifest as an increase in patient survival [50].

There are potential disadvantages to the use of video in the assessment of trauma. Assessment of the vital signs from the video recording may be difficult and an appreciation of these signs is of course important for assessing the validity and timeliness of decisions made by the trauma team. This may be overcome by a direct vital sign stream to the video or by review of the medical records. The audio quality may be poor and analysis of events outside the field of view may be difficult [44]. Errors which are better identified through medical record review include errors such as drug or fluid dosing errors (particularly important in paediatric trauma) or changes to vital signs that fail to trigger an appropriate response from the team [39].

Confidentiality issues can exist in taking and storing data about patients from whom consent is often difficult to obtain. The use of retrospective consent may be difficult, given that the patient may be sedated for some time, or moved to alternative wards no longer under the remit of the emergency department where the video was recorded. However, multiple prestigious centres across the world have employed video review as a useful, educational, quality assurance tool with the approval of legal representatives, and so long as the data is erased in a timely fashion, this should pose few problems from a legal standpoint. The assessment of video data is usually performed by an expert panel with the assistance of published guidelines; this system is time consuming and may involve subjective bias.

Furthermore, delays in analysis may lessen the potential benefits of immediate feedback. It is also costly to establish and maintain and requires routine staff participation [51].

### Simulator

Trauma team performance may be assessed using a simulator. Mannequins and simulators are increasingly being used in the assessment and education of critical care residents [52,53] and a similar approach may be appropriate in the assessment of trauma team performance.

Simulators have been used to facilitate educational goals such as communication, cooperation and leadership [54], which have already been identified as crucial qualities in trauma resuscitation [21]. A study of the use of an advanced human patient simulator (HPS) showed it to be a useful and reproducible tool for assessment of the trauma team [55], with the necessary use of video within the simulator to review team performance. Similarly, HPS has been used to demonstrate improvement in team performance following educational interventions such as an ATLS provider course or a rotation to a trauma centre. Significant improvements in critical treatment decisions, a reduced potential for adverse outcomes and improved team behaviour, function and efficiency have been observed following such interventions [55,56]. Simulators have also been used to facilitate educational on-site intervention of simulated paediatric trauma, to good effect [57]. HPS has been used to trial team behaviour assessment tools for application in trauma scenarios [58] which are thought to be important in team dynamics.

A learning curve exists in the use of simulation; the ability to interact with the simulator, 'role play' and verbalise requests for information requires some experience and this explanation may in part explain some of the improvements in team performance over time when simulation is used as the measurement tool. However, it allows exposure of the team to scenarios infrequently encountered in real life and provides a controlled, safe environment to learn from errors.

### Observation by Third Party

Observation by a third party may yield selective or biased data [59]. It is useful if just one variable or individual is being examined, for example in assessment of the performance of the team leader [42], but one or two individuals cannot be expected to review overall performance where a horizontal rather than vertical model of care is applied. The observer requires a knowledge and understanding of the processes of trauma care and needs to be available at the time of trauma calls. Although this is a resource intensive approach a 'shadow' trauma team leader is a common training technique.

### Medical Notes Review

Review of the medical notes is a slow and laborious process. Key information is often excluded from the notes [60] leading to a false negative error rate when assessing the performance of the trauma team. Essential elements of care such as the timeline, processes, communication, leadership, organisation, omissions and errors are difficult if not impossible to discern from medical record review. The contribution of professionals who do not usually enter information into the notes cannot be assessed and alternative considered diagnoses may not be recorded. For this reason the review of medical notes identifies only 20% of the errors seen on video review [39]. Furthermore, the ability to debrief, teach and learn is limited were the medical records alone are used.

## Conclusions

The rapid development of trauma services has not been universal despite the high mortality rates in the young and the repeated reporting of suboptimal outcomes. Mortality reduction requires a comprehensive performance improvement programme [61] and an effectively performing trauma team is one contributing feature of good system performance. As a component of the trauma service, the trauma team has been independently shown to reduce time in the resuscitation room, time to key investigations and to definitive care and reduce the rate of missed injury, all of which contribute to mortality reduction. If well audited, further reductions in mortality should be anticipated by education and by the introduction of processes to improve the workings of the team. Based on the limited evidence available the most effective method of trauma team audit and education appears to be by video review which can only be performed with careful consideration of consent and medicolegal issues. The use of human patient simulators may also provide a useful tool for the education of trauma team members.

## Conflicts of interests

The authors declare that they have no competing interests.

## Authors' contributions

AG and DL conceived the article concept. AG conducted the literature search and wrote the paper. DL reviewed, edited the paper and syntax.

Both authors have read and approved the final manuscript.
